# Communicative reprogramming non-curative hepatocellular carcinoma with low-dose metronomic chemotherapy, COX-2 inhibitor and PPAR-gamma agonist: a phase II trial

**DOI:** 10.1007/s12032-017-1040-0

**Published:** 2017-11-02

**Authors:** I. Walter, U. Schulz, M. Vogelhuber, K. Wiedmann, E. Endlicher, F. Klebl, R. Andreesen, W. Herr, L. Ghibelli, C. Hackl, R. Wiest, A. Reichle

**Affiliations:** 1 Health Center Alte Mälzerei, Regensburg, Germany; 2 Department Hematology and Oncology, University Hospital Regensburg, Regensburg, Germany; 3 Hospital Barmherzige Brüder Regensburg, Regensburg, Germany; 4 Health Center Regensburg, Regensburg, Germany; 5 Department of Biology, Universita’ di Roma Tor Vergata, Rome, Italy; 6 Department of Surgery, University Medical Center Regensburg, Regensburg, Germany; 7 University Hospital for Visceral Surgery and Medicine Gastroenterology, Bern, Switzerland

**Keywords:** Hepatocellular carcinoma, Biomodulatory therapy, COX-2 inhibitor, Pioglitazone, Metronomic low-dose chemotherapy, Anakoinosis

## Abstract

Systemic therapy for advanced hepatocellular carcinoma (HCC) is still challenging. A biomodulatory therapy approach targeting the communicative infrastructure of HCC, including metronomic low-dose chemotherapy with capecitabine, pioglitazone and rofecoxib, has been evaluated in patients with non-curative HCC. Altogether 38 patients were evaluable in this one-arm, multicenter phase II trial. The primary endpoint, median progression-free survival was 2.7 months (95% CI: 1.6–3.79) for all evaluable patients and 8.4 months (95% CI: 0–18.13) for patients ≥ 6 weeks on protocol. Median overall survival (OS) was 6.7 months (95% CI: 4.08–9.31) and 9.4 months (95% CI: 4.82–13.97), respectively. Most common adverse events were edemas grade 3, which were commonly related to the advanced stage, with 66% of the patients suffering from liver cirrhosis. Exploratory data analyses showed significant impact of ECOG performance status grade 0 versus 1 and CLIP score 0/1 versus > 1 on OS, 9.8 months (95% CI: 4.24–15.35) versus 2.7 months (95% CI: 1.03–4.36; *P* = 0.002), and 9.8 months (95% CI: 3.23–16.37) versus 4.4 months (95% CI: 3.14–5.66; *P* = 0.009), respectively. Preceding tumor surgery had significant beneficial impact on survival, as well as maximal tumor diameter of < 5 cm. The correlation of C-reactive protein decrease with significantly improved OS underlines the close link between inflammation and tumor control. Biomodulatory therapy in advanced HCC may be a low toxic, efficacious treatment and principally demonstrates that such approaches should be followed further for treatment of advanced HCC.

## Introduction

There is still little progress in the field of systemic therapy for non-curative hepatocellular carcinoma (HCC) despite an increasing portfolio of targeted therapies since the early 2000s [1]. As most patients diagnosed with HCC present with advanced disease, the medical need for novel systemic treatment options is high. Additionally, the incidence of HCC is rising in Europe and especially in East Asian and North African countries with 20–40 newly diagnosed cases in 100.000 inhabitants per year. Aflatoxins and infections with Hepatitis B (HBV) and C (HCV) viruses are the main risk factors in Asian and African countries [2]. Other more common risk factors for HCC in the developed countries include type 2 diabetes and obesity, besides the main risk factors of cirrhosis and chronic alcohol abuse.

Dependent on the BCLC scoring system and the Child–Pugh stage as well as the tumor mass, there are multifold therapeutic options for local treatment of HCC [3]. Sorafenib sets a novel standard in the therapy of non-curative HCC [4]. Sorafenib is a multi-tyrosine kinase inhibitor that targets the vascular endothelial growth factor receptor (VEGF-R), the platelet-derived growth factor receptor (PDGF-R) and the raf-signaling cascade [5]. Since then multiple targeted therapies have been tested and ongoing phase III trials must still confirm therapeutic improvement [6].

The present biomodulatory therapy approach aims at communicatively reprogramming both the tumor tissue and the underlying liver disease (liver cirrhosis) for attenuating HCC growth or for inducing objective tumor response. The biomodulatory approach focuses on recessive components of tumor development that means on structures and functions, which are secondarily dysregulated following oncogenic events, but which are still accessible and functionally modifiable by drug combinations with regulatory activity profiles [7, 8]. Agonists of peroxisome proliferator-activated receptors gamma (PPARγ) show a diversified regulatory activity profile in HCC, thereby attenuating in vitro HCC growth or inducing apoptosis [9]. COX-2 inhibitors may block the activity of PPARbeta, a potential tumor promotor [10]. Metronomic low-dose chemotherapy has a broad, mainly anti-angiogenic profile and showed to some degree monoactivity in advanced HCC, whereas COX-2 inhibitors and PPARγ agonists showed antitumoral activity only in the adjuvant setting [11, 12].

Metronomic low-dose chemotherapy combined with transcriptional modulators revealed broad activity in refractory hematologic or solid tumor diseases of quite different histology [7, 13]. Anti-inflammatory and anti-angiogenic activity profiles in preclinical and clinical studies suggested concerted activity of the present schedule also in up-to-now non-curative HCC.

## Patients and methods

### Patients

Patients enrolled onto the study were at least 18 years old, had histologically confirmed, locally advanced or metastatic HCC and did not qualify for further local tumor ablative procedures. Barcelona Clinic Liver Cancer (BCLC) and CLIP staging (CLIP score includes Child–Pugh stage, tumor morphology and extension, serum alfa-fetoprotein (AFP) levels and portal vein thrombosis) have been retrospectively performed. Liver cirrhosis has been histologically diagnosed. The study was conducted in accordance with Good Clinical Practice guidelines and the declaration of Helsinki and was approved by the local ethics committee (Ethics Committee of the University Hospital of Regensburg). Patients presented were recruited between February 2004 and November 2006.

### Study design and treatment

Data presented here are derived from a prospective one-arm, multicenter phase II clinical trial to assess progression-free survival (primary endpoint), safety and tolerability of a combined biomodulatory therapy.

Treatment was administered in 4-week cycles after a 2-week lead-in phase with pioglitazone and rofecoxib only. Documented in patients diaries, patients administered themselves orally metronomic low-dose chemotherapy with capecitabine (1 g/m^2^) two times daily from day 15+, the COX-2 inhibitor rofecoxib (25 mg every day without any interruption) and the PPAR-gamma agonist pioglitazone (60 mg every day without any interruption), both from day 1+.

Because of cardiac events during administration of rofecoxib for pain control, rofecoxib had been withdrawn from the market September 2004. Following study interruption, substitution of rofecoxib by etoricoxib 60 mg daily had been approved by the local ethics committee.

Treatment was continued until disease progression or permanent therapy interruption due to therapy-associated toxicities, or withdrawal of patient occurred. Patients received follow-up until death or study termination.

Adverse events (AEs) were graded according to the common terminology criteria for adverse events (CTCAE version 3.0). In case of AEs due to capecitabine the treatment was interrupted until the symptoms resolved or the severity was at maximum grade 1. During the next cycle the dose was adapted to 75% of the original dose. In case of grade 4 toxicities or a recurrent less severe AE dose reduction was adapted to 50% of the original dose. In case that a patient had a third AE under this dose adaption the patient had to go off study.

In case of a grade 1 AE due to rofecoxib/etoricoxib, the dose was reduced to 25 and 60 mg, respectively, every second day. If the AE due to the administration of the COX-2 inhibitor was ≥ grade 2, the treatment was interrupted until the symptoms resolved or the severity was at maximum grade 1.

Pioglitazone dosage was reduced to 45 mg daily in case of a grade 1 AE. If the AE was ≥ grade 2, the treatment was interrupted until the symptoms resolved or the severity was at maximum grade 1. Pioglitazone dosage could be reduced to 15 mg daily and resumed if toxicity resolved or the severity was at maximum grade 1.

### Study assessments

Screenings included medical history, physical examination, assessment of Eastern Cooperative Oncology Group (ECOG) performance status, biochemistry (kidney, liver and coagulation) and hematology, brain and bone scans (if indicated), and assessment of ongoing symptoms and adverse events (AEs); they were performed preceding study treatment, and before start of each cycle. Laboratory testing was repeated after the lead-in phase with pioglitazone and rofecoxib, thereafter, every 4 weeks and at the end of treatment or on withdrawal.

AEs were graded according to National Cancer Institute Common Terminology Criteria for Adverse Events version 3.0 and were evaluated for causality. Tumor assessments were done by computed tomography and ultrasound sonography at screening, every 8 weeks for the first 24 weeks, and every 12 weeks thereafter. Disease response was evaluated by investigators using RECIST criteria.

To estimate the anti-inflammatory activity of the treatment schedule, C-reactive protein (CRP) concentrations in serum were monitored as marker for systemic inflammation. As tumor markers serum alpha-feto-protein (AFP) and lactate-dehydrogenase (LDH) concentrations were measured at each study visit.

### Statistical analysis

Primary endpoint of the study was progression-free survival (PFS). Secondary endpoints included overall survival (OS) and safety. A one-sided, binomial hypothesis test with a target significance level *α* = 0.1 and a target power 1 − *β* = 0.80 was used for analysis. Under these assumptions, 35 patients had to be enrolled in this single-arm study plus 5 additional patients, considering a dropout rate of 15% non-evaluable patients.

Efficacy analyses, defined by protocol, were based on (1) patients, who received at least once study medication, (2) on the intent-to-treat population (ITT) that comprised all patients with ≥ 6 weeks on study, and on the per protocol population (PP), i.e., patients without any dose reduction in study drugs. Safety was analyzed in patients who received at least one dose of the study drug. PFS and OS were summarized using Kaplan–Meier estimates and were compared by log-rank test. As defined in the protocol, one-sided *P* values were calculated for OS and PFS comparisons. Cox regression was used to analyze the influence of dichotomous separated patient, disease or treatment characteristics on OS and PFS.

## Results

### Patient characteristics

Table 1 summarizes disease and demographic characteristics of all evaluable patients (*n* = 38) who were treated according protocol. Three additional patients were enrolled on study, but never received study medication due to rapid deterioration of underlying liver disease during the screening phase. These three patients were excluded from further analysis.

**Table 1:** Demographic and disease characteristics (*n* = 38)

Disease characteristics	Number of cases
Age	
< 60	15
≥ 60	23
Gender	
Male	29
Female	9
ECOG (at start of therapy)	
0	29
1	8
Tumor size (cm)	
≤ 5	19
> 5	19
Cirrhosis^a^	
Yes	25
No	13
Distant metastasis	
Yes	25
No	13
Prior surgery	
Yes	16
No	22
Prior systemic therapy	
Yes	9
No	29
Prior local ablative therapy^b^	
Yes	10
No	28
CLIP score^c^	
0	3
1	20
2	8
3	3
4	4

^a^All Child–Pugh A

^b^Different locally applied methods including transarterial chemoembolization (*TACE*), radio frequency ablation (*RFA*), ethanol ablation (*PEI*) and thermal ablation

^c^Log-rank test was performed between CLIP score groups 0 and 1 versus 2–4, *n.s.* not significant

Two patients went off study within the run-in phase (withdrawal of informed consent), six patients showed a very early progression within the first 6 weeks on study. All these patients rapidly died of progressive liver/tumor disease.

Thirty-nine percent of the patients had a CLIP score of 2–4, 66% liver cirrhosis (alcoholic 68%, virus hepatitis 32%), 66% distant metastases (lung, bone, lymph nodes) and 50% a maximum tumor diameter of > 5 cm. Half of the patients received prior systemic and/or local treatment. Two-third of the patients had metastatic tumors and three patients portal vein invasion.

### Study treatment

Evaluable study participants comprised a cohort of *n* = 38 patients, patients ≥ 6 weeks on treatment a cohort of *n* = 30 (ITT population), the PP (per protocol) population *n* = 21 patients.

The median number of treatment cycles for all evaluable patients was 3.6, for the ITT population 11.2, and for the PP population 4.5. At data cutoff, treatment was ongoing in 3 patients.

Dose reductions were necessary in nine patients, in case of rofecoxib/etoricoxib mainly due to grade 3 edemas (*n* = 4) and grade 1 renal failure (*n* = 6) (dose reduction of rofecoxib/etoricoxib and pioglitazone). Four patients had dose reduction of capecitabine due to hand-foot-syndrome.

### Efficacy

Table 2 shows the median progression-free and overall survival of the different patient cohorts. Both, the median overall and progression-free survival were best in the ITT population. Scheduled dose reduction did not impact outcome: In the PP population, the median overall survival was even shorter with 7.3 months compared to the median overall survival of the ITT population with 9.4 months.

**Table 2:** Median overall and progression-free survival in the study subpopulations

	Number of cases	Median OS [months] (95% CI)	Median PFS [months] (95% CI)
All pts included	38	**6.7**	**2.7**
		(4.08–9.31)	(1.60–3.79)
ITT	30	**9.4**	**8.4**
		(4.82–13.97)	(0–18.13)
PP	21	**7.3**	**3.4**
		(2.96–11.63)	(2.45–4.34)

A statistically significant result was suggested when the observed *P* value was less than 5% are given in bold

*ITT* (intent-to-treat): all patients treated for > 6 weeks, *PP* (per protocol): patients without dose reduction of the anti-angiogenic therapy, *OS* overall survival, *PFS* progression-free survival, 95% *CI*: 95% confidence interval

Eight patients (21%) had progressive tumor disease within 6 weeks, 28 (74%) stable disease for 2.5–36 months+. Two patients (5%) achieved objective tumor response (partial remission). In one of these patients explorative laparoscopy was performed as HCC lesions in the liver completely resolved. Histologically, however, residual disease was found. The disease control rate was 79%.

### Safety

Altogether, little severe toxicity occurred. Table 3 summarizes moderate (≤ grade II) and severe (≥ grade III) toxicities observed during the trial period in all evaluable patients. Most common were grade 3 edemas. Since more than 60% of the patients had liver cirrhosis already before start of therapy, edema had been a common problem in these patients, also before study medication. Therefore, the presence of more than 50% severe edema in the study population may be also related to progression of the underlying liver disease. Hand-foot syndromes seen in the study were related to low-dose metronomic therapy with capecitabine. In total, there had been 14 events with moderate toxicity of hand-foot syndromes (HFS) and 4 severe events requiring a dose reduction of capecitabine.

**Table 3:** Side effects of study medication

Adverse event	Grade ≤ II	% of all ≤ II AEs	Grade ≥ III	% of all ≥ III AEs
Edema	8	10.5	11	52.4
Colitis	1	1.3		
Diarrhea	8	10.5	2	9.5
Nausea/vomiting	4	5.3		
Dermatitis	1	1.3		
Hand-foot-syndrome	14	18.4	4	19.1
Mucositis/stomatitis	3	3.9		
Ascites	3	3.9	1	4.8
Leukopenia	4	5.3	1	4.8
Thrombocytopenia	2	2.6		
Anemia	8	10.5	1	4.8
Hypernatremia	2	2.6		
Fever	2	2.6		
Weight loss	1	1.3		
Anorexia			1	4.8
Elevated transaminases (ALT/AST)	3	3.9		
Renal failure	10	13.2		
Itching	1	1.3		
Nose bleeding (epistaxis)	1	1.3		
Total number	76	100	21	100

Numbers of adverse events (highest grade per patient, evaluable patients: *n* = 38)

No cardiac adverse events occurred neither during therapy with rofecoxib nor with etoricoxib.

### Explorative correlative studies

Significant correlations could be found between overall survival and dichotomously separated patient or disease characteristics, namely for ECOG status, CLIP score, preceding tumor surgery, CRP, AFP and LDH concentrations in serum at study inclusion. No corresponding correlations could be demonstrated concerning PFS.

Neither age, nor gender, presence or absence of distant metastases, or any prior systemic or local ablative therapy or the presence of liver cirrhosis had any significant impact on overall survival within the patients’ cohorts studied.

**Fig. 1:**
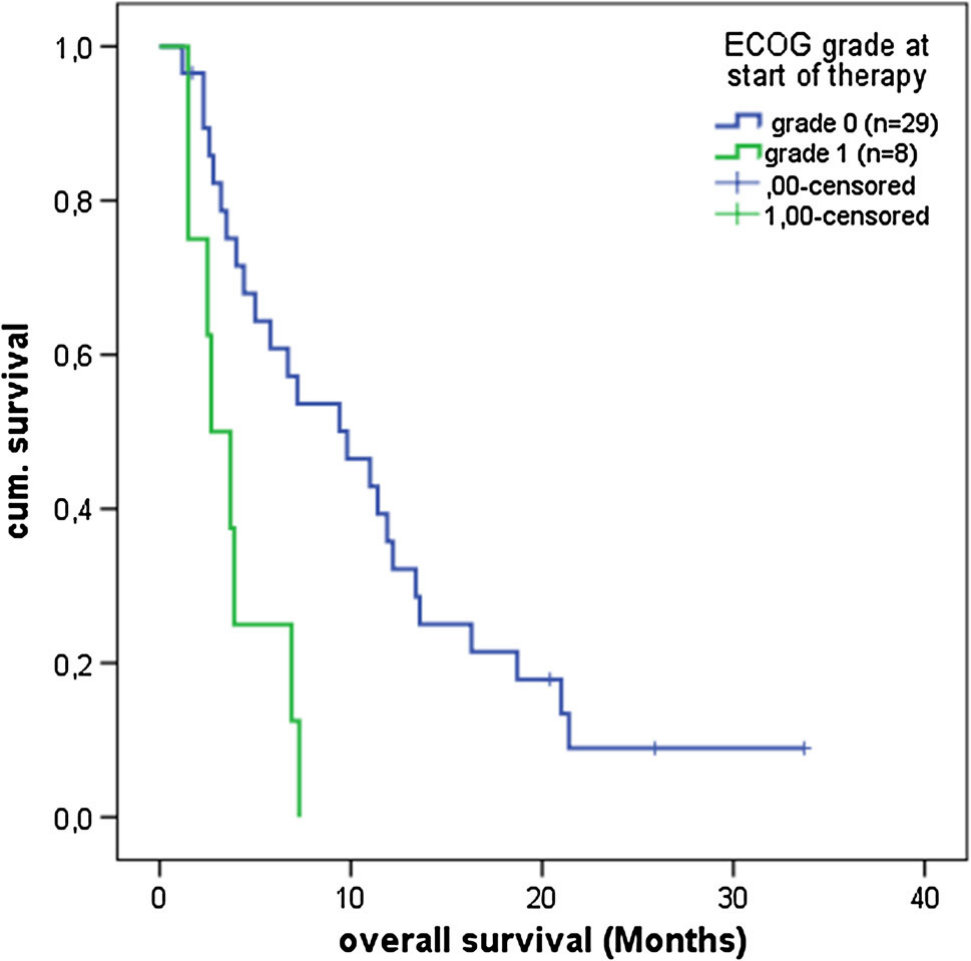
Significant correlation between ECOG grade 0 (*n* = 30) versus 1 (*n* = 8) at study inclusion and overall survival (*P* = 0.002)

*ECOG status* Patients with an ECOG status 1 (*n* = 8) at study inclusion had a median overall survival of 2.7 months (95% CI: 1.03–4.36) versus 9.8 months (95% CI: 4.24–15.35) for ECOG 0 (*n* = 29) (Fig. 1). ECOG 0 at study inclusion significantly correlated with improved overall survival (*P* = 0.002).

Fourteen of the 29 patients with ECOG grade 0 at study inclusion did not change ECOG status until end of therapy. Fifteen showed a worsening of ECOG status during treatment to ECOG 1 (*n* = 8) or ECOG 2 (*n* = 6).

*CLIP score* Patients with a CLIP score of 0 and 1 at study inclusion (*n* = 23) had a median overall survival of 9.8 months (95% CI: 3.23–16.37) versus 4.4 months (95% CI: 3.14–5.66) in patients with CLIP scores ≥ 2 (*n* = 15; *P* = 0.009). None of the other scoring systems, such as the Barcelona classification (BCLC), the Okuda score system and the international scoring system UICC showed any significant correlation with overall survival (data not shown).

### Surgery prior to enrollment had a beneficial effect on the overall survival

In all study cohorts, prior tumor surgery had significant beneficial impact on overall survival. Median OS for all evaluable patients (*n* = 38) was 5.0 months (95% CI: 1.87–8.12) in patients without prior surgery (*n* = 28) versus 9.8 (95% CI: 5.88–13.72) in the population with prior surgery (*n* = 16, *P* = 0.041). *P* values were 0.019 and 0.002 for the ITT and PP cohorts, respectively.

Since tumor size is a known prognostic parameter in resectable HCC [14], we correlated the maximum tumor size (maximum diameter of the largest tumor) obtained by computer tomography (CT scan) at start of therapy with overall survival (Table 4). In all study subpopulations patients with tumors not exceeding a maximal diameter of 5 cm had a significant longer median overall survival. With biomodulatory therapy maximum tumor size keeps its predictivity.

**Table 4:** Tumor size at study inclusion correlated with the median overall survival in the three study cohorts

	Tumor size (cm)	Number of cases	Median OS [months]	(95% CI)	*P* (log rank)
All pts	≤ 5	18	9.4	(0.00–20.21)	0.031
	> 5	20	5.0	(2.92–7.079)	
ITT	≤ 5	14	12.2	(9.45–14.95)	0.005
	> 5	15	5.8	(0.00–11.66)	
PP	≤ 5	7	13.4	(9.55–17.25)	0.022
	> 5	13	6.9	(2.79–11.01)	

Significance *P* < 0.05

*ITT* (intent-to-treat): all patients treated > 6 weeks, *PP* (per protocol): patients with dose reduction due to adverse events are excluded, *OS*: overall survival, 95% *CI*: 95% confidence interval

### Serum concentrations of CRP, AFP and LDH at start of therapy are significantly correlated with overall survival

Before start of therapy the serum biomarkers C-reactive protein (CRP), alpha-feto-protein (AFP) and lactate-dehydrogenase (LDH) concentrations in serum were measured. All these parameters are frequently elevated in HCC patients. For explorative analysis, patients were grouped according to their serum concentrations in either low or high: The cutoff for CRP in serum was ≤ 30 mg/l, for AFP ≤ 400 ng/ml and for LDH ≤ 250 U/l (Table 5). Patients with a high concentration of the respective tumor markers had significantly shorter median overall survival.

**Table 5:** Serum concentrations at study inclusion and median overall survival

Serum values prior to therapy	Means of serum values (Std.-dev.)	Number of cases	Median OS (95% CI)	*P* value (log rank)
CRP < 30 mg/l	9.87 (8.03)	22	7.2 (2.5–11.8)	0.014
CRP ≥ 30 mg/l	56.07 (29.7)	7	3.6 (0.8–6.4)	
AFP < 400 ng/ml	82.6 (114.7)	19	11.0 (8.1–13.8)	0.001
AFP ≥ 400 ng/ml	28417.1 (78987.6)	17	3.6 (2.05–5.1)	
LDH < 250 U/l	178.2 (43.5)	23	11.4 (5.3–17.5)	0.007
LDH ≥ 250 U/l	379.9 (333.6)	12	5.4 (1.5–9.3)	

Significance *P* < 0.05

*CRP* C-reactive protein, *AFP* alpha-feto-protein, *LDH* lactate-dehydrogenase, *OS* overall survival, 95% *CI* 95% confidence interval

### Serum values of CRP and AFP measured during the anti-inflammatory therapy are correlated with the overall survival

The acute phase C-reactive protein (CRP) was closely monitored during therapy as an indicator for therapy-induced inflammation control. Patients with constantly declining CRP serum values or whose values constantly were beyond the cutoff 10 mg/l were grouped together (*n* = 17). On the other hand, patients with rising CRP values during the treatment period or whose values were constantly above the cutoff 10 mg/l were grouped together as well (*n* = 11). The median overall survival of the CRP responder group was 11 months 95% CI: 2.66–19.33), whereas in the CRP non-responder group the median overall survival was significantly shorter with 3.7 months (95% CI: 2.95–4.44) (*P* = 0.005).

At study visits the tumor marker alpha-feto-protein (AFP) was measured during the treatment phase. Again, patients were grouped to a cutoff value of ≤/> 100 ng/ml and by rising or falling trends. The median overall survival of the AFP responder group (*n* = 11) was 13.6 months (95% CI: 6.58–20.61), whereas in the AFP non-responder group (*n* = 22) the median overall survival was significantly shorter with 4.4 months (95% CI: 2.46–6.33) (*P* = 0.01). Follow-up data of five patients were missing.

In contrast, no significant correlation with OS could be observed for LDH serum values measured during therapy (data not shown).

## Discussion

A disease control rate of 79% (at least stable disease) in the present single-arm phase II trial indicates that a combined biomodulatory therapy approach, including drugs with poor or no monoactivity, may be active in advanced, non-curative HCC. Efficacy cannot be delineated from the primary endpoint median PFS, which compares with the placebo arm of the SHARP study [4]. However, considering patients > 6 weeks on treatment, the median PFS compares with the sorafenib arm in the SHARP study. Since 2008 sorafenib became standard treatment of advanced HCC in patients. Recently, lenvatinib proofed to be equivalent efficacious [6].

Comparing inclusion criteria of the current study and those of the SHARP trial, many patients enrolled in the presented trial could not have been included in the SHARP trial, predominantly due to liver-associated comorbidity and systemic pre-treatment [4].

### Biomodulation seems to compare with classic targeted therapies

Median overall survival in evaluable patients is poorer than that of the placebo group in the SHARP trial: Reasons may be the heterogeneous study population with far advanced HCC and liver disease [15], extrahepatic tumor spread, high CLIP scores and frequent liver cirrhosis with consecutive liver insufficiency. The early rapid deterioration of eight patients seems to be predominantly due to the underlying liver disease and less to failure of the experimental treatment schedule.

Moreover, the disease control rate of 79% compares with data obtained by classic targeted therapies including brivanib, sunitinib, sorafenib plus erlotinib (phase III trials in first-line), or in second-line brivanib, everolimus, ramucirumab (7.6–9.4 months median OS), or immune checkpoint inhibitors (in phase II) with a disease control rate of about 70% [6, 16].

### Treatment-specific activity profile of biomodulatory therapy

Despite heterogeneous patient characteristics, compromising to some degree the comparison with published trial data, the present retrospective data analyses are indicative for a treatment-specific activity profile of the novel biomodulatory therapy approach: Outcome of the chosen biomodulatory therapy turned out to be significantly dependent on ECOG status, CLIP score, maximum diameter of the largest tumor, preceding tumor surgery, CRP and AFP concentrations in serum at study inclusion.

High CRP levels in serum at study inclusion are known predictors for inferior OS [17]. As expected from the anti-inflammatory activity profile of the present biomodulatory schedule, the study highlights CRP response as predictor for OS [18]. Anti-inflammatory response proofed to be a reliable biomarker for treatment outcome [19, 20].

Although PPAR-gamma agonists directly regulate alpha-feto-protein expression, decreasing AFP levels in serum is predictive for significantly improved OS [15].

Noteworthy, the treatment schedule is active irrespectively of the presence/absence of distant metastases or prior systemic/local ablative therapy or decrease/increase of LDH concentrations in serum during follow-up. No significant impact of extrahepatic metastases on overall survival contradicts experiences with classic targeted therapies, as genetic heterogeneity at metastatic sites normally implicates differential response to targeted therapies [21]. Biomodulatory therapies aim at attenuating or normalizing tumor-promoting hallmarks of cancer, such as pro-inflammatory and pro-angiogenic processes in the tumor environment irrespectively of expected genetic heterogeneity. All genetic or molecular-genetic constellations lastly maintain convergent constituted hallmarks of cancer [7].

The observations that the presence of liver cirrhosis is no discriminating parameter concerning OS, and that a therapy-associated decrease of the unfavorable prognostic parameter, CRP concentration in serum, is significantly associated with improved OS, suggest the assumption that the administered biomodulatory therapy may induce anakoinosis that means, decisively reprograms tumor-promoting functions provided by the cirrhotic liver and neighboring tumor microenvironment [7]. That absence of liver fibrosis may overcome tumor size as prognostic parameter supports the suggestion [22]. However, maximum tumor size, a prognostic parameter for OS in resectable HCC, remains a prognostic parameter in unresectable, non-curative HCC [14].

Failure to demonstrate prognostic parameters for PFS again indicates that outcome parameters such as PFS are additionally affected by the tumor-initiating liver disease and, therefore, no useful primary endpoints [23].

### Mechanisms of action

All combinatorial administered drugs have been excessively studied at least in preclinical settings [6, 24, 25]. COX-2 expression in tumor and non-tumor liver disease plays a crucial role during carcinogenesis and propagation of HCC. COX-2 inhibitors act pro-apoptotic and anti-proliferative [26]. Experimentally, celecoxib suppressed cancer stemness and progression of HCC via activation of PPARγ and up-regulation of PTEN [27]. COX-2 and peroxisome proliferator-activated receptor delta are involved in important growth promoting signaling pathways in human hepatocellular carcinoma [28].

PPARγ is heterogeneously expressed in HCC cells [29]. Corresponding agonists act growth inhibitory, pro-apoptotic via up-regulation of tumor suppressor genes, e.g., PTEN, or a putative tumor suppressor gene, the growth differentiation factor-15 [30] and promote accumulation of p27(Kip1) [31]. PPARγ agonists induce expression of CITED2 in HCC cell lines and epigenetically regulate microRNA-122 [32]. The PPARγ agonist rosiglitazone may inhibit HCC cell growth by blocking the oncogenic function of septin 2 (SEPT2) [33]. One possible mechanism of action of PPARγ agonists in HCC is the regulation of the deregulated Wnt pathway via PTEN [34]. Pioglitazone-related up-regulation of PTEN further sensitizes the HCC cell lines to 5-fluorouracil (5-FU) [35, 36], which is the active metabolite of capecitabine. PPARγ agonists may directly inhibit proliferation of hepatic stellate cells and consecutive hepatic fibrosis [37].

As hypervascularization is a major characteristic of HCC, anti-angiogenic therapy schedules are promising treatment elements of non-curative stages in HCC [38]. Metronomic low-dose chemotherapy boosts CD95-dependent anti-angiogenic effect of the thrombospondin expression [39], while PPARγ agonists may promote expression of the receptor for thrombospondin, CD 36 [40]. Metronomic capecitabine in advanced hepatocellular carcinoma patients has already shown clinical activity [41].

### Toxicity

The adverse events that were more common are mainly mild to moderate in severity. The two most frequent adverse events in this study are edema and hand-foot syndrome. Edemas are related to both the underlying hepatic disease (liver cirrhosis) and to therapy with pioglitazone and rofecoxib. The incidence of hand-foot syndrome related to capecitabine is much lower than expected with a conventional dosing of capecitabine [42]. The overall tolerability of the schedule is indicated by the small proportion of patients, which received scheduled permanent dose reduction of single therapy elements.

## Conclusion

In summary, this study shows that a biomodulatory therapy approach may be efficacious and tolerable in advanced HCC. As expected from preclinical data, clinical activity of a biomodulatory therapy approach gives hints that the combination of predominantly regulatory active therapy elements may induce anakoinosis, namely communicatively reprograms on three levels, the tumor cells, adjacent stroma cells and the tumor-promoting environment, i.e., liver cirrhosis [7]. Biomodulation lends itself as novel therapy element in addition to classic targeted therapies. Efficacious anti-inflammatory therapy might partially overcome sorafenib resistance [43].
